# Factors Affecting the Choice of a Clinician for Orthodontic Treatment in Malaysia

**DOI:** 10.7759/cureus.70574

**Published:** 2024-09-30

**Authors:** Ahmed Said Elsayed Ahmed Elagamy Moussa, Umi Mardhiyyah Mat Ali, Liyana Ghazali

**Affiliations:** 1 School of Dental Sciences, Universiti Sains Malaysia Health Campus, Kota Bharu, MYS; 2 Orthodontic Unit, School of Dental Sciences, Universiti Sains Malaysia Health Campus, Kota Bharu, MYS

**Keywords:** factors influencing choice of clinician, general dental practitioner, orthodontics, patient's preference, specialist orthodontist

## Abstract

Introduction

In Malaysia, orthodontic treatments are provided by general dental practitioners (GDPs) or specialist orthodontists. Evidence suggested that the public could be confused about clinicians providing orthodontic treatments. The objectives of this study were to assess the public understanding of the difference between a specialist orthodontist and a GDP who provides orthodontic treatment and to evaluate the factors that might influence the choice of a practitioner.

Methods

An online, dual-language survey comprising 185 Malaysian adults was completed. Thirteen questions regarding social demographics, knowledge of clinicians providing orthodontic treatment, and factors that influence the choice of a clinician were asked.

Results

Around 74% of Malaysian respondents knew the differences between a specialist orthodontist and a GDP providing orthodontic treatment. Being treated by a specialist orthodontist and the cost of treatment were identified as the most important factors in choosing an orthodontic service provider followed by recommendations from GDP, location, testimony, and lastly recommendations from friends/family.

Conclusion

The majority of Malaysian respondents could identify the difference between a specialist orthodontist and a GDP who provides orthodontic treatment. Being treated by a specialist orthodontist and cost were the main factors that influenced the choice of a clinician providing orthodontic treatment in Malaysia.

## Introduction

A general dental practitioner (GDP) better known as a dentist provides oral healthcare to the public. A GDP examines and diagnoses patients while administering treatments, such as tooth restorations, dental extractions, dentures, scaling, and emergency care. A GDP may refer patients who need further complex care to the appropriate specialist. To qualify as a dental surgeon, one must obtain a degree in dental surgery and complete a compulsory service in the government sector before being certified as a dentist [1]. Subsequently, a specialist orthodontist is a scientific title that a dentist obtains after completing at least three years of additional formal, structured, clinical orthodontic education [2].

Orthodontics is defined as the branch of dentistry that is concerned with the growth of craniofacial complex and the development of teeth and occlusion, including the diagnosis, interception, and treatment of anomalies associated with the occlusion [3]. Class I malocclusion is the most common feature followed by Class II and Class III. Cenzato et al. found that crowding is the most common trait of malocclusion with a prevalence of up to 84% [4]. Increased severity of malocclusion has been associated with a negative impact on oral health-related quality of life [5]. In Malaysia, orthodontic treatments may be performed by either a GDP or a specialist orthodontist.

Despite the widespread use of information technologies and the internet, evidence suggests that the public can be confused about a GDP who provides orthodontic treatment with a specialist orthodontist [6]. Some complex cases and growing patients might require planning and treatment from a specialist orthodontist for optimal results [7]. For example, cases with vertical, anteroposterior, and transverse problems, impacted teeth, or cases involving multidisciplinary treatments [8]. Marques et al. suggested that despite the advanced technology, specialist orthodontists still obtain better treatment outcomes in a shorter time as compared to GDPs [9].

Few studies have been conducted globally to explore these topics [6,10]. However, no evidence was found within the Malaysian population. Thus, this study aimed to explore and evaluate the choice of a clinician for orthodontic treatment in Malaysia and the factors that might influence the choice.

## Materials and methods

This study was approved by Jawatankuasa Etika Penyelidikan (Manusia) (JEPeM) Universiti Sains Malaysia (USM) under approval number USM/JEPeM/22040208. The sample size was calculated based on a previous study where 74% of the respondents believed an orthodontist was the most qualified person to perform an orthodontic treatment [10]. A sample size of 116 was obtained for the test with a significance level of 0.05 and a power of 0.8.

An online, dual-language questionnaire for this survey was adapted from Wishney et al. [10]. The English version of the questionnaire was translated to Bahasa Malaysia (BM) by a language expert. The BM version of the questionnaire was translated back to English followed by a pilot study to ensure the content validity. The dual-language questionnaire was made available online via Microsoft Forms (Microsoft Corp., Redmond, USA). The link to the questionnaire was distributed via email and WhatsApp groups. Before answering the questionnaire, participants were required to give consent to take part in the survey. This study included subjects from the Malaysian population aged 18-60 years. All responses and data were anonymous and stored on secured servers. Because the surveys could not be submitted without completing all questions, no incomplete data were collected.

## Results

The quota for the survey was reached within 14 days of invitations. The final sample was 185 responses, and all samples were included in the data analysis. The average time taken to complete the questionnaire was 7.2 minutes.

This questionnaire was divided into three categories. In category 1, six questions were asked regarding the demographic of the respondents. In category 2, six questions were asked regarding the choice of a clinician providing orthodontic treatment, and in category 3, respondents were asked about the factors that influenced their choice of a clinician.

Category 1

Table 1 shows the demographic data of Malaysian respondents. The majority of the respondents were female (77.8%) and belonged to younger ages, that is, 18 to 30 years. More than 80% of our respondents held at least a bachelor's degree and 65% resided in the city. Despite having a higher education level, almost half of the respondents (47.6%) had a household income of less than RM 5000, which falls into the lower B40 (bottom 40%) category based on Malaysian household income [11]. About one-third of the respondents were from Pantai Timur, while the rest of the respondents were equally distributed all across Malaysia.

**Table 1:** Overview of demographics of Malaysian respondents

Demographic	No	Percentage (%)
Gender		
Male	41	22.2
Female	144	77.8
Age (years)		
18-30	163	88.1
31-40	11	5.9
41-50	5	2.7
52-60	6	3.3
Level of education		
High-school	11	5.9
Diploma	14	7.6
Bachelors degree	147	79.5
Masters/PhD	13	7.0
Household income		
Less than RM 4849	88	47.6
RM 4850-10960	56	30.3
More than RM 10961	41	22.1
Region		
City	120	64.9
Rural	65	35.1
Region of Malaysia		
Klang Valley (Kuala Lumpur, Selangor, Negeri Sembilan)	31	16.8
Pantai Timur (Kelantan, Terengganu, Pahang)	67	36.2
Selatan Malaysia (Johor, Melaka)	24	13.0
Utara Malaysia (Kedah, Perak, Pulau Pinang, Perlis)	41	22.2
East Malaysia (Sabah, Sarawak, Labuan)	22	11.8

Category 2

**Figure 1:**
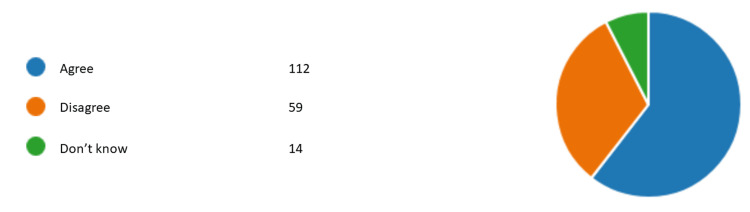
Response to question 7 Question 7: In Malaysia, a GDP who performs orthodontic treatment must also be a specialist orthodontist. GDP: general dental practitioner

**Figure 2:**
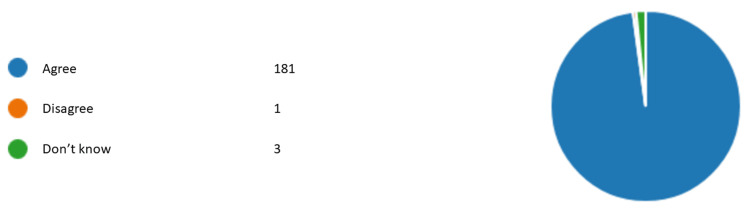
Response to question 8 Question 8: In Malaysia, a specialist orthodontist must also hold qualification as a GDP. GDP: general dental practitioner

**Figure 3:**
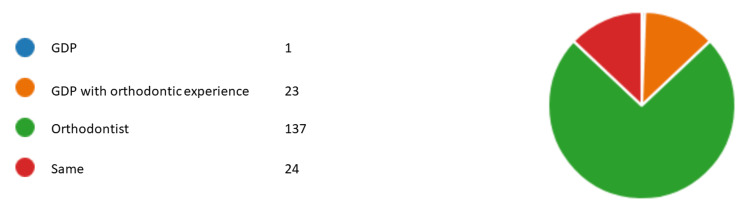
Response to question 9 Question 9: The most qualified clinician to perform orthodontic treatment. GDP: general dental practitioner

**Figure 4:**
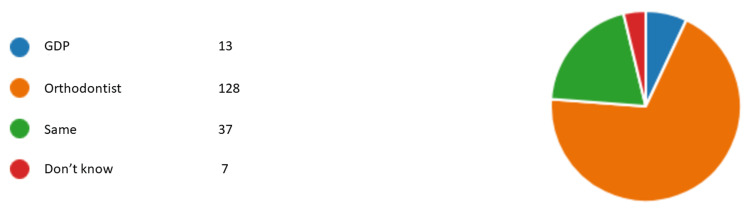
Response to question 10 Question 10: More training time and university education. GDP: general dental practitioner

**Figure 5:**
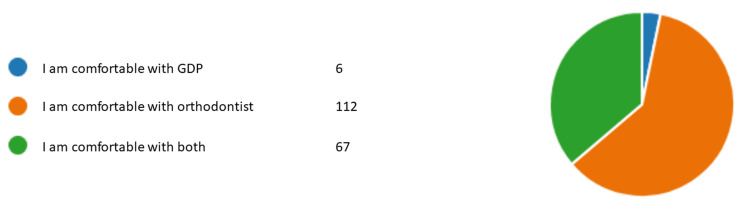
Response to question 11 Question 11: Statement that best describes how you feel. GDP: general dental practitioner

**Figure 6:**
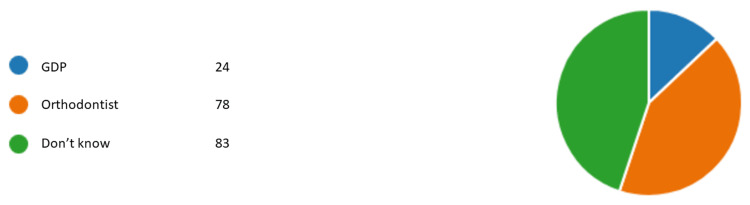
Response to question 12 Question 12: If you or a family member have had orthodontic treatment, who performed it? GDP: general dental practitioner

Figures 1-6 represent the results for category 2. In question 7, respondents were asked whether the dentist performing orthodontic treatment must be a specialist orthodontist. Around 60.5% (n= 112) agreed, 31.8% (n= 59) disagreed, and 7.5% (n= 14) did not know (Figure 1). In question 8, almost all of our respondents agreed that a specialist orthodontist must also qualify as a general dentist (Figure 2). In question 9, 74% of our respondents thought that specialist orthodontists were more qualified to perform an orthodontic treatment. About 12.4% of the respondents thought general dentists with experience in orthodontics were more qualified to perform orthodontic treatment than specialist orthodontists themselves (Figure 3). Figure 4 demonstrates the results for question 10, where 70% of respondents agreed that in terms of training time specialist orthodontists require more university education than GDPs. Figure 5 demonstrates the results for question 11. Around 60% of our respondents were more comfortable with specialist orthodontists performing orthodontic treatment. Only about 4% were more comfortable with a GDP performing their orthodontic treatments. Despite that, 45% of respondents did not know whether a GDP or specialist orthodontist performed orthodontic treatment on them or their family members (Figure 6).

Category 3

**Figure 7:**
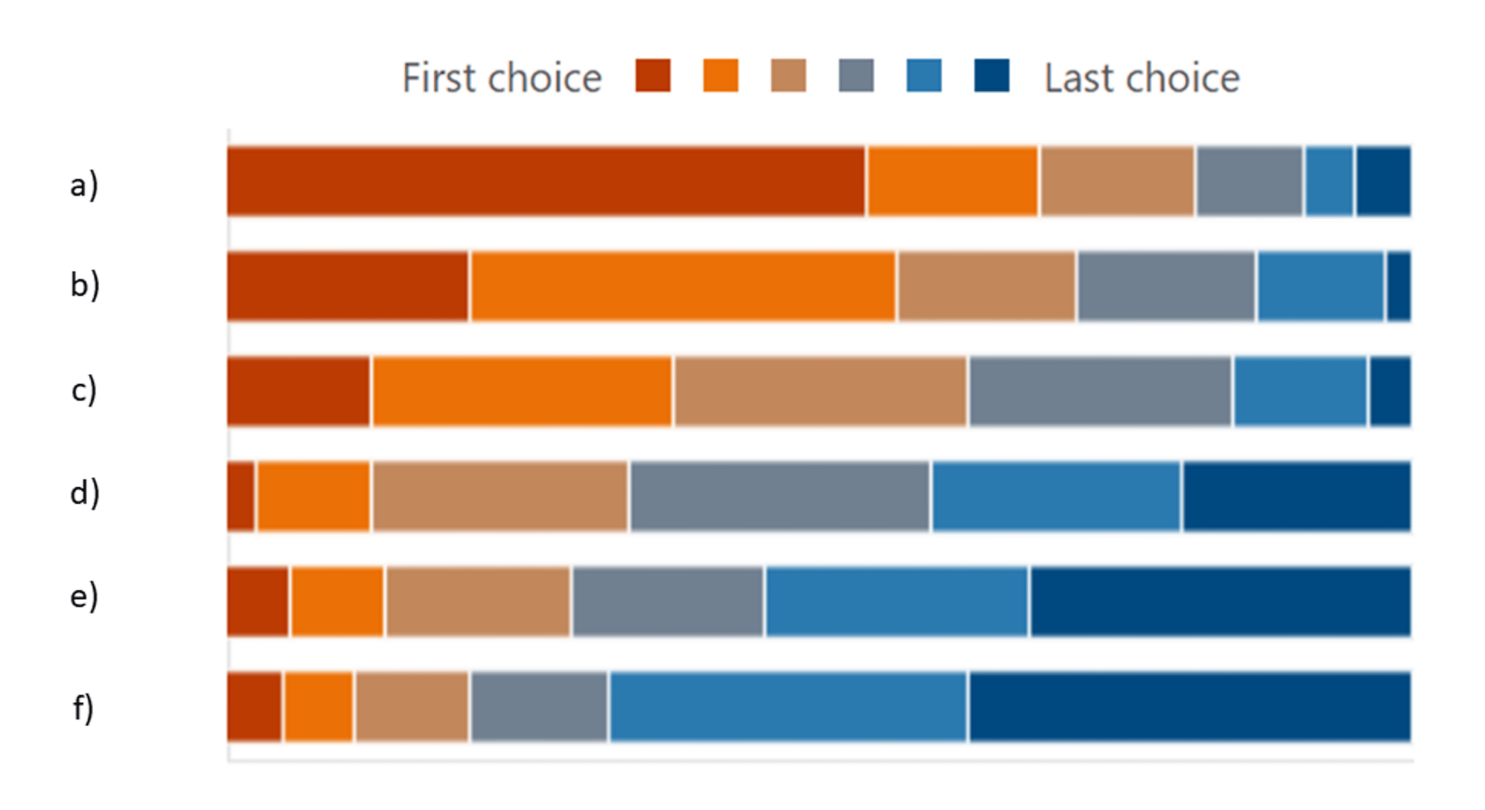
Factors influencing the choice of a clinician providing orthodontic treatment Options: (a) Whether the practitioner is a specialist orthodontist; (b) Cost of treatment; (c) Recommendation from general dental practitioner; (d) Location of procedure; (e) Testimony; (f) Recommendation from friends/family

Figure 7 demonstrates the factors that influenced the respondent's choice of a clinician providing orthodontic treatment. There were six factors included, and our respondents ranked “Whether or not the practitioner is a specialist orthodontist” as the most important factor followed by “Cost,” “Suggestion from referring dentist,” and “Suggestion from friends/relatives." “Location” was ranked the least important factor when choosing a clinician to perform orthodontic treatment.

## Discussion

In our survey, we evaluated the understanding of the adult Malaysian population of different clinicians providing orthodontic treatments. We also explored the factors that might influence the choice of a clinician providing orthodontic treatment. Increasing demand for orthodontic treatments inspires clinicians to explore more regarding this topic further.

The majority of our respondents were females (78%) and young adults (88%) who were more synonymous with orthodontic treatment. Young female patients are found to have more desire and willingness to receive orthodontic treatment [12]. Young adult orthodontic patients are defined as those who miss treatment during the growing stage or those whose optimal treatment can be carried out only after cessation of growth [13]. This finding correlates with the finding by Nelson et al. that the highest number of internet and social media users among orthodontic patients is in the age group 25-34 years and more predominantly females [14]. These individuals might be more interested in orthodontic information and treatment, and thus, represent this survey's largest number of respondents.

Questions in category 2 summarized the knowledge of the Malaysian population regarding specialist orthodontists and GDPs providing orthodontic treatment. About 74% of Malaysian respondents agreed that specialist orthodontists were more qualified to perform orthodontic treatment. This finding is identical to responses found by Wishney et al. [10]. The study reported that 73.8% of Australian and 73.6% of Swedish respondents identified specialist orthodontists as more qualified than GDPs to perform orthodontic treatment. This indicates that a major proportion of Malaysian respondents recognized the expertise of specialist orthodontists. A total of 70% of Malaysian respondents knew that a specialist orthodontist requires more education than a GDP. This finding contradicts Park et al. who found that 64.2% of American respondents failed to recognize that an orthodontist requires more education than a general dentist [6]. Interestingly, despite recognizing the expertise of a specialist orthodontist, 40% of the Malaysian population is comfortable with GDPs performing their orthodontic treatment. Evidence showed that patients perceive treatment by a GDP would be more convenient and less costly [15].

In our survey, we listed six factors that might influence the choice of a clinician providing orthodontic treatment. More than half of our respondents (54.1%) thought that being treated by a specialist orthodontist was the most important factor. A similar finding was also reported by Park et al. [6]. A study conducted in Richmond, USA, found that specialist orthodontists were the preferred clinicians especially when patients are looking for higher treatment quality [16]. The second most important factor in choosing a clinician providing orthodontic treatment was cost. At least 56% of respondents ranked cost as the most and second most important factor when choosing a clinician providing orthodontic treatment. In Malaysia, the state government subsidizes orthodontic treatment for children below 18 years old, where cost might not be the main concern. The respondents in our survey, mainly young adults, had to pay for their orthodontic treatments. As reported earlier, 47.6% of our respondents had a low household income. Only 2.2% of the respondents thought cost to be their least priority when choosing a clinician providing orthodontic treatment. Many studies also reveal that the cost of orthodontic treatment is one of the most significant factors when considering orthodontic treatments [17,18].

The third most important factor was a recommendation from a general dentist. Tuncer et al. conducted a survey in Turkey and found that up to 84.6% of parents seeking orthodontic treatment were mainly based on recommendations by dentists [19]. A GDP is perceptibly responsible and becomes the main source of awareness and information regarding orthodontic treatment [6]. Location was the fourth most important factor ranked by our respondents. Due to longer treatment duration and regular appointments during the course of orthodontic treatment, location was also considered an important factor by our respondents. Warpe et al. reported that 90% of patients wanted a dental clinic that was at least an average distance from their home or office, although no specific distance was mentioned [20]. This finding, however, contradicts a recent systematic review conducted by Tee et al. [21]. The study found that the location of a dental clinic does not affect patients’ decisions when choosing their clinician and patients are willing to travel a long distance to receive treatment from their preferred clinician.

Recommendations from friends/family and testimony were ranked the least and second least important factors in choosing a clinician providing orthodontic treatment, respectively. Despite the widespread use of social media in promoting healthcare services, these two factors do not play as important roles as other factors mentioned earlier. This is in accordance with a previous study that reported 90.3% of parents do not focus on dentists’ online reviews before choosing a dentist for their children [21,22].

This cross-sectional study was conducted online using convenient sampling method. The study was limited to individuals who had access to the internet and might have a negative impact on the generalisability of the findings. In this study, we divided samples according to six regions to make sure the samples are representative of Malaysian population. The findings in this study should be interpreted with caution. In this study, we included all samples aged 18-60 years and received more interest in the younger age group. Future research could segregate patients into different age groups and include pediatric and teen patients who are more synonymous with orthodontic treatment.

## Conclusions

In conclusion, the majority of Malaysian respondents (74%) were able to identify the specialist orthodontist and general dental practitioner providing orthodontic treatment. For many people, the most important factors that influenced the choice of a clinician providing orthodontic treatment were being treated by a specialist orthodontist and cost. Other important factors included recommendations from GDPs and location. Recommendations from family/friends and testimony were less important when choosing a clinician providing orthodontic treatment.
